# Association of Value of Fibrosis-4 Index with Long-Term Mortality in Patients with Heart Failure: A Comparison Study with Fibrosis-5 Index

**DOI:** 10.34172/jcvtr.33476

**Published:** 2026-06-30

**Authors:** Halit Emre Yalvaç, Fatih Enes Durmaz, Bektaş Murat, Selda Murat

**Affiliations:** ^1^Department of Cardiology, Eskişehir City Hospital, Eskişehir, Turkey; ^2^Department of Cardiology, Muş State Hospital, Muş, Turkey; ^3^Department of Cardiology, Eskişehir Osmangazi University Hospital, Eskişehir, Turkey

**Keywords:** Fibrosis index, Heart failure, Liver fibrosis, Oedema, Platelet

## Abstract

**Introduction:**

Fibrosis-4 (FIB-4) and fibrosis-5 (FIB-5) indices are non-invasive biomarkers originally developed for hepatic fibrosis but may reflect systemic pathophysiological changes related to adverse cardiovascular prognosis. We aimed to investigate whether baseline FIB-4 and FIB-5 values predict long-term mortality in patients with heart failure (HF).

**Methods:**

This retrospective observational study included 593 patients with HF who had available long-term mortality data. FIB-4 and FIB-5 indices were calculated at admission. Patients were followed for all-cause mortality, and outcomes were evaluated at 1-year, 3-year, and 5-year intervals. Clinical, laboratory, and echocardiographic parameters were compared between survivors and non-survivors.

**Results:**

During follow-up, 1-year, 3-year, and 5-year mortality rates were 24.6%, 39.0%, and 49.7%, respectively. Non-survivors had significantly higher FIB-4 and FIB-5 values compared with survivors (*P*<0.001 for both). ROC curve analyses demonstrated that both indices had significant predictive performance for long-term mortality. Optimal cut-off values showed good sensitivity and specificity for identifying high-risk patients.

**Conclusion:**

Higher FIB-4 and FIB-5 values are associated with greater long-term mortality risk in HF patients. These indices may serve as practical, cost-effective prognostic biomarkers for mortality risk stratification in routine clinical care.

## Introduction

 Heart failure (HF) is a chronic and progressive condition characterized by the inability of the heart to maintain sufficient cardiac output to meet the body’s metabolic demands. This results in impaired functional capacity and recurrent hospitalizations due to worsening symptoms. Globally, HF is among the leading causes of mortality, and its prevalence rises significantly with advancing age in the general population.^1^ A typical consequence of HF is systemic venous congestion and reduced tissue perfusion, which contribute to hepatic dysfunction and abnormalities in liver function tests. These alterations are linked with increased levels of hepatic fibrosis markers.^2^

 The interplay between the severity of HF and liver function has recently drawn increasing attention. Venous congestion, resulting from elevated end-diastolic pressures, has been associated with hepatocellular atrophy and perisinusoidal edema, changes that can ultimately progress to liver fibrosis.^3^ Although several serum-based fibrosis indices have been associated with adverse outcomes in HF patients, the most suitable marker in this clinical setting remains uncertain.

 The Fibrosis-4 (FIB-4) index—calculated using age, aspartate aminotransferase (AST), alanine aminotransferase (ALT), and platelet count—has long been utilized to evaluate liver fibrosis, particularly in patients with viral hepatitis B or C. Beyond hepatology, it has also emerged as a reliable prognostic tool in cardiovascular medicine, predicting mortality and hospital readmission in HF patients.^4^

 More recently, the Fibrosis-5 (FIB-5) index was introduced, incorporating albumin, alkaline phosphatase (ALP), AST/ALT ratio, and platelet count. Initially validated in patients with hepatitis C virus (HCV) infection, subsequent studies have suggested that FIB-5 may have comparable or superior diagnostic performance to FIB-4 in assessing hepatic fibrosis in chronic liver diseases.^5^ While FIB-4 is increasingly considered a surrogate marker of venous congestion severity and prognosis in HF, data on the prognostic role of FIB-5 in this population are scarce. Moreover, direct comparisons of FIB-4 and FIB-5 in patients with HF have not previously been performed.

 Therefore, the aim of this study was to evaluate and compare the prognostic value of FIB-4 and FIB-5 indices in patients with heart failure with reduced ejection fraction (HFrEF).

## Materials and Methods

###  Study Design and Population

 This was a retrospective, dual-center cohort study conducted at Eskişehir Osmangazi University Faculty of Medicine and Eskişehir City Hospital. The study included patients who were hospitalized with a diagnosis of heart failure with reduced ejection fraction (HFrEF) between January 2015 and December 2023. Patients younger than 18 years of age, those with active infection, known chronic liver disease, malignancy, severe renal impairment (eGFR < 30 mL/min/1.73m^2^), or incomplete clinical data were excluded.

###  Data Collection

 Demographic data, comorbidities, laboratory test results, echocardiographic measurements, and clinical outcomes were extracted from hospital records. Laboratory parameters included aspartate aminotransferase (AST), alanine aminotransferase (ALT), albumin, alkaline phosphatase (ALP), platelet count, and other routine biochemical variables at admission.

###  Echocardiographic Evaluation

 Right ventricular function was evaluated using tricuspid annular plane systolic excursion (TAPSE) and right atrial diameter (RAD). RV dysfunction was defined as TAPSE < 16 mm. Other echocardiographic parameters such as left atrial dimension (LAD), left ventricular ejection fraction (LVEF), and systolic pulmonary artery pressure (sPAP) were also recorded.

###  Calculation of FIB-4 and FIB-5 Indexes

 The FIB-4 index was calculated in the following formula:

**FIB− 4** = age (years) × AST (U/L)/[ALT (U/L)1/2 × PLT count (109/L)]

 The FIB-5 index was determined using the following parameters: albumin, alkaline phosphatase (ALP), AST/ALT ratio, and platelet count, as previously described⁶.

**FIB-5**: [albumin (g/L) × 0.3 + PLT count (109/L) × 0.05] − [ALP

 (U/L) × 0.014 + AST to ALT ratio × 6 + 14].

###  Study Endpoints and Follow Up

 The primary endpoint was defined as 5-year all-cause mortality. Secondary endpoints included 1-year all-cause mortality and hospital readmissions. Mortality data were obtained from hospital records and, when necessary, from national death registry databases.This study was conducted in accordance with the Helsinki Declaration and was approved by the Ethics Committee of our University. The informed consent was waived due to the study design that it was retrospective.

###  Statistical Analysis

 Continuous variables with a normal distribution were expressed as mean ± standard deviation (SD), while those without a normal distribution were described as median (25th–75th percentile). Continuous variables were compared using the Student’s t-test or Mann-Whitney U test as appropriate. Categorical variables were reported as counts and percentages and compared using χ^2^ or Fisher’s exact tests. To assess cardiovascular outcomes receiver-operating characteristic (ROC) curve analysis was performed for the FIB-4 and FIB-5. The optimal cutoff value was defined as the point maximizing the Youden index ( = max [sensitivity + specificity − 1]). The study group was subsequently divided into two groups based on the ROC curve cutoff values for FIB scores. Cumulative event rates were assessed using Kaplan-Meier analysis and compared with log-rank tests. P-values < 0.05 were considered statistically significant. IBM SPSS Statistics 21.0 software (IBM Corp. Released 2012. IBM SPSS Statistics for Windows, Version 21.0. Armonk, NY, US; IBM Corp.) was used for the analyses.

## Results

 In this dual center retrospective study we enrolled 593 patients with heart failure retrospectively. The mean age was 70. 0 (61. 0-77.0) with male predominant 415 (70.0%). The mean fallow up was 5 years. According comorbidities 439 (74.0 %) of the patients had Coronary artery disease (CAD), 266 (44.9 %) had diabetes mellitus (DM) and 395 (66.6 %) of the population has hypertension (HT). Baseline characteristics of the study population were divided into two groups according the cut-off value of the FIB-5 index. Older patients were associated with lower FIB-5 level while there was difference between comorbidities and FIB-5 level. According the laboratory findings lower FIB-5 was associated with higher NTProBNP 5109 (1885-14160) vs 3631 (1433-10510), (*P* = 0.003) and lower FIB-5 was associated with lower PLT 173 (146-206) vs 249 (204-306) (*P* < 0.001). according to echocardiographic findings lower FIB-5 was associated with higher left atrial dimension and higher Estimated PAP (mmHg) [ 47.20 (42,0-50,0) vs 45.0 (39.10-4820), (*P* = 0.042) and 50,0 (40,0-60,5) vs 47,5 (35,0-60,0), (*P* = 0.046)] respectively. As Table 1 shows the baseline characteristics of the study population lower FIB-5 was associated with 1 year, 2 years and five years all- cause mortality.

**Table 1:** Patient Baseline Characteristics

	**All population**	**Low FIB-5**	**High FIB-5**	***P*** ** value**
	**(n=593)**	**(n=297)**	**(n=296)**	
Age (years)	70.0 [61.0-77.0]	71.0 [63.0-78.0]	69.0 [60.0-77.0]	0.007
Male, n (%)	415 (%70.0)	200 (%67.3)	215 (%72.6)	0.16
Coronary artery disease, n (%)	439 (%74.0)	219 (%73.7)	220 (%74.3)	0.871
Diabetes, n (%)	266 (%44.9)	132 (%44.4)	134 (%45.3)	0.84
Hypertension, n (%)	395 (%66.6)	205 (%69.0)	190 (%64.2)	0.212
Smoking, n(%)	164 (%27.7)	78 (%26.3)	86 (%29.1)	0.447
Prescription, n (%)				
Loop diuretics	318 (%53.6)	162 (%54.5)	156 (%52.7)	0.653
ACE-I/ARB	420 (%70.8)	209 (%70.4)	211 (%71.3)	0.807
Beta-blockers	556 (%93.8)	277 (%93.3)	279 (%94.3)	0.618
MRA	294 (%49.6)	143 (%48.1)	151 (%51.0)	0.485
Laboratory Data				
Haemoglobin (g/dL)	12.4 ± 2.0	12.4 ± 2.0	12.3 ± 2.1	0.606
Platelet counts	205 (163-261)	173 (146-206)	249 (204-306)	< 0.001
Albumin (g/dL)	3.7 (3.2-4.0)	3.6 (3.2-4.0)	3.7 (3.2-4.0)	0.269
ALT (U/L)	18.0 (12.0-31.0)	15.0 (11.0-26.5)	22.0 (12.5-36.0)	< 0.001
AST (U/L)	22.0 (15.0-35.0)	22.0 (15.0-35.0)	21.5 (15.0-34.0)	0.609
ALP (U/L)	81.0 (62.0-105.0)	80.0 (63.0-108.0)	82.0 (60.0-102.0)	0.648
Creatinine (mg/dL)	1.2 (0.9-1.7)	1.2 (0.9-1.8)	1.1 (0.9-1.7)	0.104
Blood urea nitrogen (mg/dL)	29.0 (20.0-44.0)	31.4 (20.0-45.9)	27.0 (20.0-40.3)	0.017
Sodium (mEq/L)	138.0 (134.0-141.0)	138.0 (135.0-141.0)	138.0 (134.0-141.0)	0.768
Potassium (mEq/L)	4.4 (4.1-4.9)	4.4 (4.1-4.8)	4.5 (4.0-4.9)	0.336
NT-proBNP (pg/mL)	4407 (1650-11439)	5109 (1885-14160)	3631 (1433-10510)	0.003
Echocardiographic Data				
Left atrial dimension (LAD) (cm)	46.0 (41.0-50.0)	47.20 (42.0-50.0)	45.0 (39.10-4820)	0.042
LVEF (%)	25.0 (18.0-30.0)	25.0 (18.0-30.0)	25.0 (18.0-31.0)	0.707
sPAP (mmHg)	50.0 (40.0-60.0)	50.0 (40.0-60.5)	47.5 (35.0-60.0)	0.046
LVDD (mm)	57.0 (51.0-62.0)	57.0 (51.5-62.0)	57.0 (51.0-62.0)	0.147
LVDS (mm)	46.0 (39.5-52.0)	46.0 (40.0-52.0)	46.0 (39.0-52.5)	0.341
1 year all-cause mortality, n (%)	146 (%24.6)	84 (%28.3)	62 (%20.9)	0.038
3 year all-cause mortality, n (%)	231 (%39.0)	136 (%45.8)	95 (%32.1)	< 0.001
5 year all-cause mortality, n (%)	295 (%49.7)	178 (%59.9)	120 (%40.5)	< 0.001

ACE-I: Angiotensin converting enzyme inhibitors, ALP:Alkaline phosphatase, ALT: Alanine transaminase, ARB: Angiotensin receptor blockers, AST: Aspartate transaminase, Fib-4: Fibrosis-4, Fib-5: Fibrosis-5, MRA: Mineralocorticoid Receptor Antagonist, NT-proBNP: N-terminal pro b-type natriuretic peptide, LVDD: Left ventricle left ventricle diastolic dimension, LVDS: Left ventricle left ventricle systolic dimension, LVEF: Left ventricle ejection fraction, sPAP: systolic pulmonary artery pressure.

 Receiver operating characteristic (ROC) curve analysis was performed to compare the predictive performance of the FIB-4 and FIB-5. Fibrosis-5 was predicted the 5 years all-cause mortality with as sensitivity of 44.3 % and specificity of 76.9 % (95% CI 0.559-0.640; *P* < 0.001), and the cut-off value was -12.01 (Figure 1).

**Figure 1:**
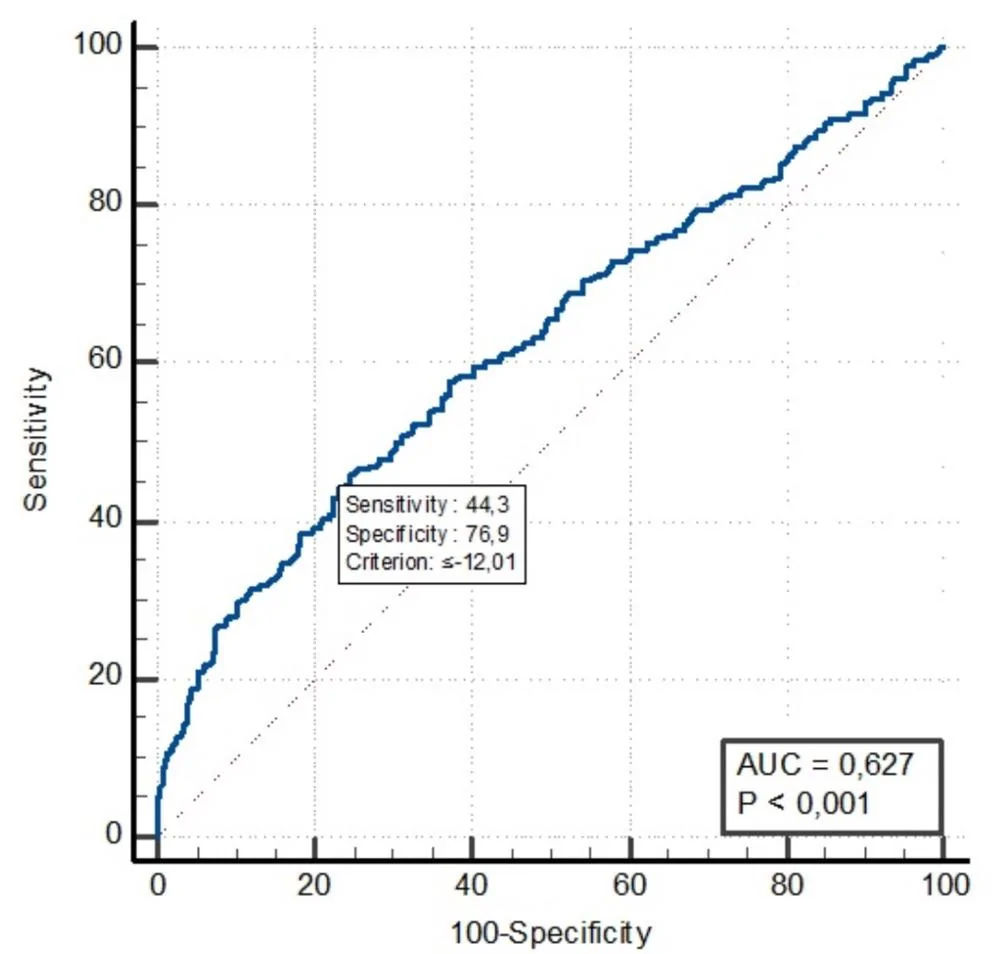
ROC curve analysis of the FIB-5 index for predicting 5-year all-cause mortality in patients with HF.

 In pairwise analysis, we compared the ROC curves of the FIB-4 and FIB-5 for 5- year all-cause mortality in whole patients there was no significant differences between FIB-4 and FIB-5 (0.600 vs 0.627, *P* = 0.198) (Figure 2). After separating patients by the age ≤ 75 years. The C-statistics of FIB-5 was significantly greater than that of the FIB-4 in patients aged ≤ 75 years (0.115 and 0.662, *P* = 0.0417) (Figure 3).

**Figure 2:**
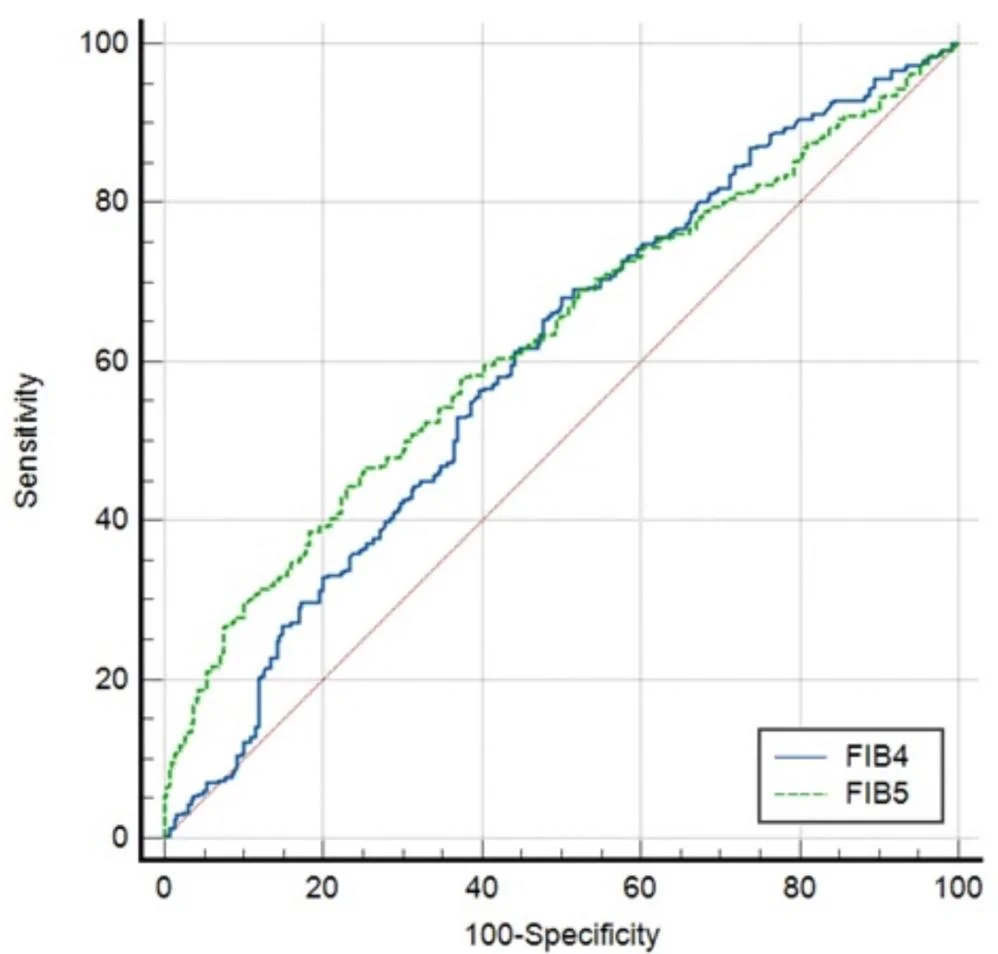
Pairwise comparison of ROC curves for the FIB-4 and FIB-5 indices in the overall study population.

**Figure 3:**
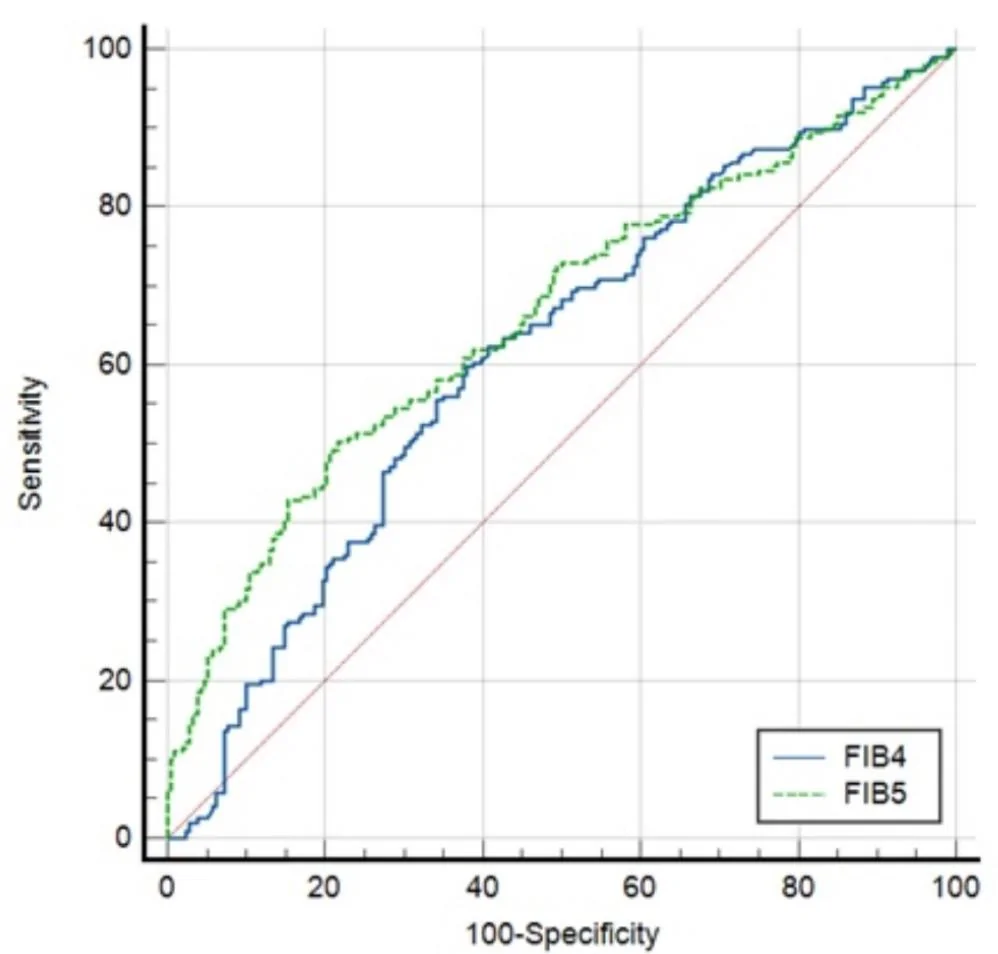
ROC curve comparison of the FIB-4 and FIB-5 indices in patients aged <75 years.

 The univariate and multivariate logistic regression analysis of the patients are presented in Table 2. In the multivariate analysis, FIB-5, left atrial dilatation (LAD) and systolic pulmonary artery pressure (sPAP) were identified as independent predictors of long-term all-cause mortality in patients with HFrEF.

**Table 2:** Univariate and Multivariate Logistic Regression Analyses Predictors of Mortality

	**Univariate Analysis**		**Multivariate Analysis**	
**Baseline Characteristics**	**OR(95%CI)**	***P*** ** Value**	**OR(95%CI)**	***P*** ** Value**
Age	1.02 (1.01-1.04)	< 0.001	1.01 (0.99-1.03)	0.077
FIB-4 index	1.02 (0.98-1.07)	0.263		
FIB-5 index	0.91 (0.88-0.94)	< 0.001	0.92 (0.88-0.96)	< 0.001
Gender(female)	0.86 (0.60-1.22)	0.415		
Hypertension	1.55 (1.10-2.19)	0.012	1.42 (0.93-2.16)	0.099
Diabetes	1.32 (0.95-1.83)	0.088		
Coronary artery disease	1.20 (0.83-1.74)	0.313		
LVEF (%)	0.97 (0.95-0.99)	0.014		
LAD (cm)	1.06 (1.04-1.09)	< 0.001	1.03 (1.00-1.06)	0.033
RAD (cm)	1.04 (1.01-1.07)	0.002		
sPAP (mmHg)	1.02 (1.01-1.03)	< 0.001	1.01 (1.00-1.02)	0.025
Hemoglobin (g/dL)	0.92 (0.85-0.99)	0.037		
Albumin (g/dL)	0.78 (0.60-1.02)	0.077		
Creatinine (mg/dL)	1.14 (0.99-1.32)	0.064		

Fib-4: Fibrosis-4, Fib-5: Fibrosis-5, LAD: Left atrial dilatation, LVEF: Left ventricle ejection fraction, OR: Oid ratio, RAD: Right atrial dilatation, sPAP: systolic pulmonary artery pressure.

 RV dysfunction, defined as TAPSE < 16 mm, was identified in 147 patients (24.8%). These patients had significantly higher sPAP values and larger right atrial dimensions compared with those without RV dysfunction. When stratified according to RV function, low FIB-5 values remained significantly associated with long-term all-cause mortality, whereas the prognostic value of FIB-4 was attenuated. In multivariable analysis including RV function, FIB-5 persisted as an independent predictor of mortality, suggesting its prognostic value irrespective of RV dysfunction.

## Discussion

 In this study, we investigated the prognostic role of two widely used non-invasive liver fibrosis indices, FIB-4 and FIB-5, in patients with HFrEF and provided a head-to-head comparison of their predictive performance. Our findings demonstrated that both indices were significantly associated with long-term all-cause mortality. However, the FIB-5 index emerged as a stronger and more consistent predictor, retaining its prognostic significance even after adjustment for conventional risk factors and right ventricular dysfunction. These observations support the potential utility of FIB-5 as a more reliable biomarker of adverse outcomes in HFrEF.

 The pathophysiological background linking HF and liver function is complex and multifactorial. In patients with advanced HF, chronic venous congestion leads to elevated central venous pressure, sinusoidal dilatation, hepatocellular atrophy, and perisinusoidal edema, which ultimately progress to fibrosis. In parallel, reduced cardiac output contributes to impaired hepatic perfusion and ischemic injury. Several studies have reported that abnormalities in liver function tests, including elevated transaminases and cholestatic enzymes, are correlated with the severity of HF and with poor outcomes.^7-9^ Non-invasive indices such as FIB-4 and FIB-5, originally developed for staging fibrosis in viral hepatitis or other chronic liver diseases, have therefore been repurposed in recent years as surrogate markers of cardio-hepatic interaction in HF populations.^10^

 Our analysis adds to this growing body of evidence by directly comparing FIB-4 and FIB-5 in a large HFrEF cohort. While FIB-4 has been extensively validated and is easy to calculate, its discriminative ability in cardiovascular disease has been moderate.^11^ FIB-5, by incorporating albumin and ALP in addition to platelet count and AST/ALT ratio, provides information on hepatic synthetic capacity and cholestatic changes, both of which may reflect chronic venous congestion more accurately.^12^ This may explain the superior prognostic performance of FIB-5 in our cohort.

 An important aspect of our results is the relationship between liver fibrosis indices and right ventricular dysfunction. RV failure is a well-established driver of morbidity and mortality in HF, largely through its impact on systemic venous pressures and hepatic congestion.^13^ In our study, RV dysfunction was present in approximately one-quarter of the patients, a prevalence consistent with previous reports. Importantly, FIB-5 remained a significant predictor of mortality even after adjustment for RV dysfunction, suggesting that it captures prognostic information beyond echocardiographic markers of right heart performance.^14^ This independence enhances its potential value in clinical decision-making, particularly in situations where echocardiographic assessment of RV function is limited or inconclusive.

 From a clinical perspective, the use of easily available laboratory-based indices such as FIB-4 and FIB-5 offers several advantages. These scores require no specialized imaging or invasive procedures, can be calculated at the bedside, and may be repeated over time to track disease progression.^15^ Incorporating FIB-5 into routine risk stratification of HFrEF patients could therefore help clinicians identify high-risk individuals who may benefit from closer monitoring, optimized medical therapy, or earlier consideration of advanced treatments such as device therapy or transplantation.

 Our findings should also be viewed in the context of other non-invasive markers of fibrosis. Prior work has evaluated scores such as the NAFLD fibrosis score and APRI in HF, with variable prognostic performance.^16-18^ Systematic reviews have suggested that FIB-5 may outperform these indices in chronic liver disease, but data in cardiac populations remain limited. By providing a direct comparison with FIB-4, our study contributes novel insights and highlights FIB-5 as a promising candidate for broader validation in HF populations.^19^

 This study has several limitations. First, it was conducted at two centers, and a multicenter design would be required to improve generalizability. Second, liver biopsy, the gold standard for assessing fibrosis, was not performed, and our analysis relied entirely on non-invasive indices. Imaging modalities such as abdominal ultrasound, CT, or MRI were also not systematically used, which limited our ability to distinguish whether hepatic impairment was primarily due to intrinsic liver disease or secondary to HF-related congestion. Third, clinical details regarding the presence of congestive symptoms (e.g., peripheral edema, hepatomegaly) and biomarkers of inflammation or ischemia (e.g., C-reactive protein, troponin, natriuretic peptides) were not available for inclusion in our models. Furthermore, liver elastography (Fibroscan), which provides a direct and quantitative measure of hepatic stiffness, was not performed. Finally, invasive hemodynamic evaluation with right heart catheterization was not available in patients with venous congestion, precluding definitive assessment of whether hepatic fibrosis was due to primary hepatic pathology or secondary to HF-related hemodynamic derangements.

## Conclusion

 In conclusion, both FIB-4 and FIB-5 indices were significantly associated with long-term mortality in patients with HFrEF. However, FIB-5 demonstrated superior and independent prognostic value, even after adjustment for right ventricular dysfunction and other established risk factors. These findings suggest that FIB-5 may serve as a more reliable, accessible, and cost-effective tool for risk stratification in HFrEF, complementing conventional clinical and echocardiographic evaluation. Future prospective, multicenter studies are warranted to validate these results and to further explore the role of non-invasive fibrosis indices in guiding the management of patients with heart failure.

## Competing Interests

 The authors declare that they have no competing interests.

## Ethical Approval

 This study was conducted in accordance with the principles of the Declaration of Helsinki. The study protocol was reviewed and approved by the Ethics Committee of Eskişehir Osmangazi University (Approval date: 22.03.2022; Approval number: 43). The requirement for informed consent was waived due to the retrospective design of the study.
